# Depicting People in Visual Cues Affects Alcohol Cue Reactivity in Male Alcohol-Dependent Patients

**DOI:** 10.3390/brainsci12030307

**Published:** 2022-02-24

**Authors:** Abdulqawi Alarefi, Xunshi Wang, Rui Tao, Qinqin Rui, Guoqing Gao, Ying Wang, Liangjun Pang, Chialun Liu, Xiaochu Zhang

**Affiliations:** 1 Department of Radiology, The First Affiliated Hospital of USTC, School of Life Science, Division of Life Science and Medicine, University of Science & Technology of China, Hefei 230027, China; bmeeng@mail.ustc.edu.cn (A.A.); wy1987@ustc.edu.cn (Y.W.); cllustc@ustc.edu.cn (C.L.); 2 Affiliated Psychological Hospital of Anhui Medical University, Hefei Fourth People’s Hospital, Anhui Mental Health Center, Hefei 230017, China; 11318281@zju.edu.cn (X.W.); taor@ahmu.edu.cn (R.T.); qinr@ahmhcentre.com (Q.R.); 2013203020049@whu.edu.cn (G.G.); 3 Centers for Biomedical Engineering, School of Information Science and Technology, University of Science & Technology of China, Hefei 230027, China; 4 Department of Psychology, School of Humanities & Social Science, University of Science & Technology of China, Hefei 230026, China

**Keywords:** alcohol, craving, cue reactivity, fMRI, sensorimotor

## Abstract

Cue reactivity is often used to study alcohol cues brain responses. Standardized image sets are used, but the effect of viewing people interacting with the alcohol drink remains unclear, which is associated with the factors of alcohol cues that influence the degree of response to alcohol stimuli. The present study used fMRI to investigate the reactivity of alcohol dependence (AD) inpatients to alcohol cues with or without human drinking behavior. Cues with a human interacting with a drink were hypothesized to increase sensorimotor activation. In total, 30 AD inpatients were asked to view pictures with a factorial design of beverage types (alcoholic vs. non-alcoholic beverages) and cue types (with or without drink action). Whole-brain analyses were performed. A correlation analysis was conducted to confirm whether the whole-brain analysis revealed cue-related brain activations correlated with problem drinking duration. The left lingual gyrus showed significant beverage types through cue type interaction, and the bilateral temporal cortex showed significant activation in response to alcohol cues depicting human drinking behavior. The right and left lingual gyrus regions and left temporal cortex were positively correlated with problem drinking duration. Sensorimotor activations in the temporal cortex may reflect self-referential and memory-based scene processing. Thus, our findings indicate these regions are associated with alcohol use and suggest them for cue exposure treatment of alcohol addiction.

## 1. Introduction

Increased cue reactivity, as well as increased craving and drinking urges in response to alcohol-associated cues, characterizes AD. Alcohol craving, defined as a persistent or strong desire for alcohol, is also a critical factor in substance-related disorders. It can be caused by exposure to an object or environment that promotes alcohol consumption [1]. During the development of AD, the effects of alcohol gradually become associated with drinking behavior [1], a phenomenon known as cue reactivity. Exposure to stimuli (cues) can elicit conditioned responses accompanied by substance consumption along with craving [2]. Neural reactivity to alcohol-related stimuli has been comprehensively explored and found to be vital in developing and maintaining alcohol addiction [3].

According to current neuroimaging data, salient substance stimuli, compared with neutral control stimuli, given to substance users evoke increases in activity across the mesocorticolimbic and reward circuits [1,4,5,6,7]. Heavy alcohol usage is known to affect different synaptic structures and neurotransmitter systems within these circuits, resulting in deficits in motivational drive and hypersensitive conditioned responses to alcohol-related cues, such as the cue-induced desire for alcohol consumption [8]. Alcohol-related stimuli trigger brain responses in the reward system, which are mostly responsible for one’s incentive salience or “desire” for alcohol. Increased motivational salience of alcohol cues, as indicated by their influence on the mesocorticolimbic circuitry, is a key component of alcohol-induced neuropathology, according to these lines of evidence [1]. Positive associations between substance brain responses and measures of substance-induced cravings, attentional bias, eye movements, dependency severity, and relapse have been documented often [9,10]. Additionally, the brain representations of reward values of substance cues and the motivational processes of incentive salience that drive substance-seeking behavior are expected to be reflected in these substance cue-evoked responses [3]. The effect of substance-associated modulation of the mesocorticolimbic circuitry was suggested to extend to sensory representations of substance stimuli that improve the sensory representations responses in the temporal, occipital, and parietal structures [11,12]. This was supported by a recent voxel-wise meta-analysis of AD fMRI studies that reported significant alcohol cue-induced activation in AD patients, including the bilateral middle occipital gyrus, right inferior occipital gyrus, right superior parietal gyrus, right angular gyrus, and right posterior cingulate [3], suggesting their involvement in mediating multiple aspects of alcohol-dependent behaviors development and maintenance.

Despite such studies using similar experimental designs and analyses, the activation patterns were not always consistent. This can be partly attributed to a limited understanding of the factors that modulate the degree of cue-reactivity effects. To address this issue, a recent study decomposes substance cues into drug, drug use action, and drug use tools, and used these cues to induce distinct brain activity [13]. The study clearly demonstrates that drug use action can also induce sensorimotor areas, which might associate with automatic drug-seeking behavior. Based on this, one could also expect cue-related brain regions would correlate with measures of habitual behaviors. In addition, certain factors, such as the contents of the cue images, alcohol preferences, and sample size, were also found to affect cue effect [14,15]. Moreover, as the contents of stimuli may affect the level of craving induced and the magnitude of cue reactivity, some research groups have developed standardized alcohol-related pictures for alcohol-related research (e.g., [16]). Different types of stimuli have been used in some studies, such as simple images with/without brand information, depictions of different settings (e.g., bar, restaurant, home), or pictures of people holding or interacting with alcoholic drinks [5,17,18]. Finally, it remains unclear how much of this factor is attributed to different results.

The dual-process model of addiction [2] emphasizes that implicit cognitive processes can further bias attention toward incentive cues. The attentional bias processing toward substance-related cues is fostered by the hypersensitivity to the motivational effects of substance incentive [19]. Therefore, the drinking environment that contains drinking behavior is likely associated with the cues and further amplifies the urge to consume alcohol. For instance, Lee et al. [20] found that the self-reported craving from heavy drinkers was greater in response to an alcoholic beverage in a drinking context (e.g., toasting) than the one in a blank background. Their results imply that cues involving the drinking context seem to be more effective in provoking an urge to drink. Previous neuroimaging studies reported that substance-related cues involving drinking actions elicit stronger brain responses in the sensorimotor-related regions in addition to the reward-related regions [4]. For instance, in addition to activations in the reward-related regions, cues with drug-consuming actions have also induced alterations in motor preparation regions, such as the posterior central gyrus, superior parietal lobule, and inferior parietal lobule [21,22]. Moreover, it has long been suggested that the activation of conditioned sensorimotor associations is another route through which substance cues may induce increased substance use [23]. In addition, it was previously reported that the sensorimotor cortex is also an essential neural basis of automatic substance-using behavior and craving, and may mediate substance-seeking behavior and craving [13].

Specifically, it was previously suggested that the frontoparietal mirror neuron system might be disparate nodes or modules of a self-referential processing system, which is important in substance abuse and addiction [24]; that is, observing action could increase the likelihood that a person will perform these actions [25]. This can be supported by several studies. Neuroimaging studies have shown that seeing related actions can activate brain regions that are not common reward-related regions in cue-reactivity effect, such as the inferior parietal lobule and the premotor cortex [26,27], and bilateral middle temporal cortex, bilateral calcarine, and bilateral inferior parietal cortex [13]. The eliciting of automatized action representations may carry out substance-seeking and substance-taking actions [12]. Therefore, self-referential processing can be triggered by action/use contexts, where the action-context image may stoke the self-referential circuitry [28].

Furthermore, little is known about the factors associated with the alcohol cues that influence the degree of response to alcohol cues and craving [29]. It is difficult to determine from previous studies which particular element of the alcohol cue causes the specific brain region reactivity, and which area’s activity serves as the cue’s underlying mechanism, such as cues depicting human drinking actions. In addition, studies have suggested that automatic substance-seeking behavior could facilitate certain aspects of cue processing (e.g., sensorimotor processing of substance cues [13,30]). Although previous studies have reported findings from human brain imaging, there is still a need to study the effect of depicting human drinking actions in alcohol cue reactivity.

The main objective of the present study is to explore the brain responses elicited by visual alcohol cues with/without a person interacting with the drink. We hypothesize that the cues with human drinking behavior will find enhanced brain activations in the sensorimotor regions. Specifically, we expect that the alcohol cues depicting drinking behavior will find strong activation in sensorimotor regions (e.g., bilateral middle temporal cortex and inferior parietal gyrus). In addition, mesolimbic and prefrontal regions related to reward are also expected to show significant activations. Additionally, based on the brain region identified above, we further investigate if the activities from these regions would correlate with certain behavioral measures such as the duration of problem drinking.

## 2. Materials and Methods

### 2.1. Participants

In total, 30 men with AD (mean age, 39.6 years, with an age range of 25 to 56 years) were recruited from Anhui Mental Health Center at Hefei Fourth People’s Hospital. At the time of the study, all of the patients were under an inpatient treatment protocol. All the patients met the criteria for AD syndrome as their primary diagnosis, according to the International Classification of Disease, 10th edition (ICD-10: F102). Before the experiment, patients were abstinent from alcohol for 2–3 weeks (mean, 25 days; range, 15–44 days) and were not allowed to consume alcohol in the center. Inclusion criteria were those (a) aged 18–55 years old; (b) meeting the DSM-IV diagnostic criteria for AD, without acute alcohol withdrawal syndrome (stop drinking for 14–30 days), and having a CIWA-Ar score < 7; (c) having no significant contraindications for MRI scan. Exclusion criteria were patients (a) with any neurological or mental disorders or dependent on substances other than alcohol or nicotine, (b) with a history of brain injury, and (c) taking drugs with significant anticholinergic effects such as benzodiazepine and clozapine. Inclusion and exclusion criteria followed a previous study [31]. The severity of alcohol dependence was measured using the Alcohol Use Disorders Identification Test (AUDIT) [32]. The duration of problem drinking was assessed by asking each participant, “Calculate the years of problematic drinking.” To confirm the reliability of the scores, a family member of each participant was asked a similar question at the admission time of the treatment program. Written informed consent was obtained from all patients. Subjects were compensated for their participation in the experiment with non-alcoholic beverages or cigarettes. The study was approved by the Ethics Committee of Hefei Fourth People’s Hospital. Further details are given in the online Supplementary Materials (File S1, Subjects’ details).

### 2.2. Cue-Reactivity Task

**Figure 1:**
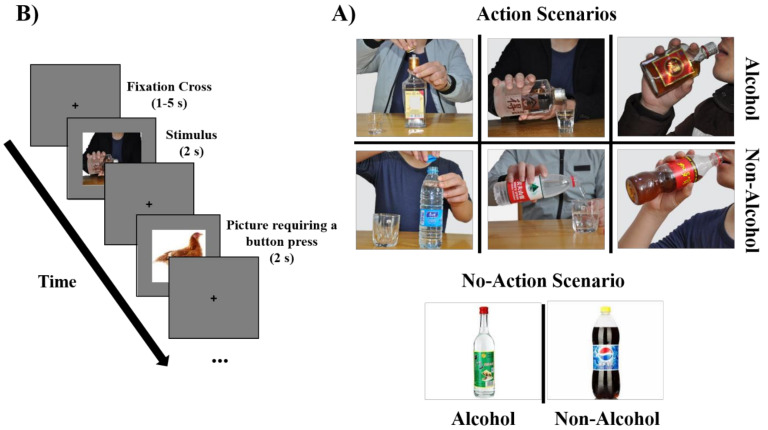
Experimental structure and design: (**A**) each experiment has two types of beverage cues (alcohol and matched non-alcohol with different scenarios) and animal cues. The design of the task was single trial, in which each trial contains a fixation cross presented for 1–5 s before the stimulus image of an alcohol or non-alcohol cue was presented for 2 s. The animal picture was used to require pressing a button to keep the subject’s attention on the task. (**B**) sample task stimuli. The top six images show examples of alcohol/non-alcohol cues as a function of the action scenario. The bottom two images show examples of alcohol/non-alcohol cues as a function of the no-action scenario.

We used an event-related design fMRI task. Each participant completed 2 runs of a visual cue-reactivity task [33]. In each run, participants viewed 90 cues (Figure 1B): 40 alcohol cues with action and no-action scenarios (e.g., a bottle and full glasses against a plain background, hands opening a bottle, a person pouring an alcoholic drink, and a person drinking); 40 matched action and no-action scenarios depicting non-alcoholic beverages; 10 pictures of animals. In each run (Figure 1B), each cue was presented for 2 s in a pseudorandom order. Pseudorandom intervals were generated using a function in MATLAB, in which each run contains the same number of alcohol/non-alcohol cues and action/no-action cues. Between each picture, a fixation cross was presented for an interval ranging from 1 to 5 s (Mean = 3 s). The duration of each run was 7.5 min. A 1 min rest was given to the participants after each run. To keep the patients’ attention on the task, they were requested to press a button when an animal picture appeared on the fMRI display. The animal pictures were evenly inserted into each run randomly.

### 2.3. Cue Design

The cues consisted of 40 alcohol-related pictures matched with 40 non-alcohol-related pictures. Alcohol and non-alcohol pictures were matched for size and visual complexity. All pictures were first selected based on common brands and beverages in China. A rating procedure was conducted with another group of AD patients to ensure the cues were suitable for the patients. The results of the rating procedure showed a significant difference in craving scores between the alcohol and the non-alcohol pictures (Supplementary Materials, File S1).

Faces in the cues were generally cropped out to control for any potential confounding effects [34], with the exception that the mouth was kept to avoid unnaturalness in some pictures, such as when hands were holding drinks near the face. Each image resolution was adjusted to 700 × 450. The online Supplementary Materials shows further details of cue design and the matching of alcohol/non-alcohol and action/no-action pictures (Supplementary Materials, File S1).

### 2.4. Data Acquisition

MRI scans were acquired at the Medical Sciences Building, University of Science and Technology of China, on a 3T scanner (Discovery MR750 system, General Electric Health Care, Milwaukee, WI, United States), equipped with an 8-channel head coil. Functional scans were obtained by a T2*-weighted echo-planar imaging sequence with the following parameters: echo time (TE) = 30 ms, repetition time (TR) = 2000 ms, flip angle = 90°, field of view (FOV) = 192 × 192 mm^2^, matrix = 64 × 64, voxel size 3 × 3 × 3 mm^3^, and 36 slices per volume with whole-brain coverage. Anatomical images were collected with a gradient-recalled scanning sequence with the following parameters: TR = 8.16 ms, TE = 3.18 ms, FOV = 256 × 256 mm^2^, voxel size 1 × 1 × 1 mm^3^, and 188 sagittal slices.

### 2.5. Image Processing

Functional MRI data were processed with the SPM toolbox (SPM12; http://www.fil.ion.ucl.ac.uk/spm (accessed on 20 May 2021)). To avoid the non-equilibrium effects of magnetization, the first two volumes were removed. Next, a slice-timing correction was applied to adjust the effect of interleaved scanning. Spatial realignment (i.e., rigid body correction) was then conducted to the functional volumes for head movement through realignment to the mean fMRI image. The motion exclusion criteria relied on the mean framewise displacement of >0.25 mm for all subjects. Images were then normalized using EPI templates into the Montreal Neurological Institute (MNI) space, with an isotropic 3 × 3 × 3 mm^3^ voxel size. The normalized images were then smoothed with an 8 × 8 × 8 mm^3^ Gaussian kernel. The data were high-pass filtered using a high-pass temporal filter with T = 128 s, which was incorporated into the first-level analysis.

### 2.6. Statistical Analyses

Statistical analyses of fMRI data were performed using Statistical Parametric Mapping (SPM12; http://www.fil.ion.ucl.ac.uk/spm (accessed on 20 May 2021)). To observe the blood-oxygen-level-dependent (BOLD) response to each condition, first-level analysis was performed for each participant by convolving the reactions to each type of cue with the canonical hemodynamic response function. Reactions to cues depicting alcoholic and matched non-alcoholic beverages were modeled as separate event types. The four main trial types were no-action alcohol, action alcohol, no-action non-alcohol, and action non-alcohol. Stimuli requiring a button press (catch trials) were modeled as a regressor of no interest, and rigid body corrections were modeled as covariates of no interests. The resulting contrasts of all subjects were then used as input for second-level analysis. The main effect of alcohol was further tested by a one-sample *t*-test between images of alcoholic beverages and images of non-alcoholic beverages. A 2 × 2 repeated-measures analysis of variance was employed using beverage (alcohol, matched non-alcohol) × cue type (action, no-action) as within-subject factors. Post hoc analysis was conducted by paired *t*-tests based on the activated voxels that survived the familywise error (FWE) correction to determine the response changes between conditions. Post hoc multiple comparisons were Bonferroni corrected at *p* < 0.05.

Pearson correlations were performed between the problem drinking years and the mean response in brain activation clusters revealed by whole-brain analysis, including right temporal cortex, left temporal cortex, left parahippocampal gyrus, and left lingual gyrus. The masks of the clusters were created using xjView (https://www.alivelearn.net/xjview/ accessed on 30 May 2021), in which four masks in total were created. Then, the averaged BOLD signal (beta values) from the alcohol versus matched non-alcohol and action versus no-action contrasts were extracted per individual using MarsBaR (http://marsbar.sourceforge.net/ accessed on 30 May 2021). Bonferroni correction was applied for multiple comparisons.

## 3. Results

### 3.1. Behavioral Data

Demographic and clinical variables are shown in Tables S1–S5 (Supplementary Materials, File S1) of the 30 participants, 25 finished the AUDIT questionnaire because some subjects did not score all the AUDIT questionnaire items and were then excluded from the analysis. The mean AUDIT score was 26.20 (SD = 6.70), and the score was significantly higher than zero, *t*(24) = 19.33, *p* < 0.001. The mean duration of problem drinking years was 12.43 (SD = 3.13), which was also significantly higher than zero, *t*(29) = 21.01, *p* < 0.001.

### 3.2. fMRI Results

#### 3.2.1. The Main Effect of Beverage Types

Parameter estimates under the four experimental conditions were extracted from the activated clusters and submitted to a two (cue category) by two (cue type) repeated-measures ANOVA. We identified the brain regions associated with the main effect of cue category (i.e., type of beverage; Supplementary Materials, File S1 (Figure S3 and Table S5)).

#### 3.2.2. The Main Effect of Cue Types

**Figure 2:**
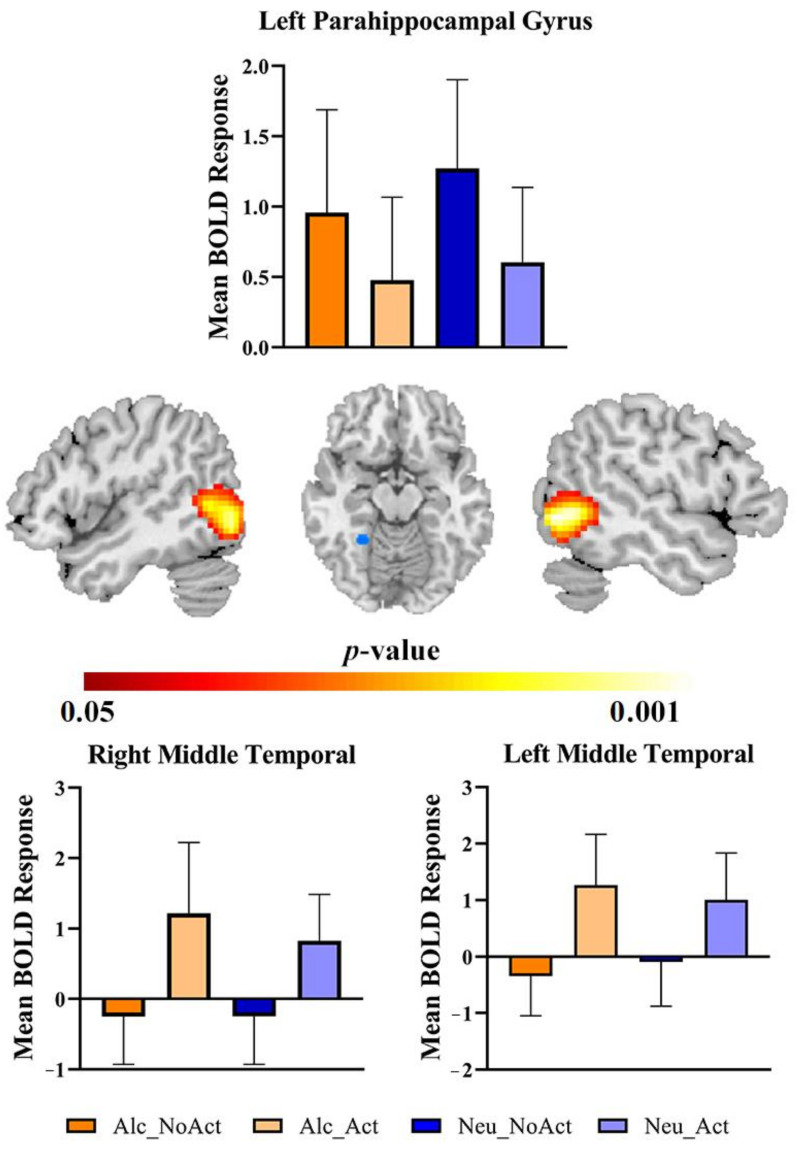
Brain activations of cue type main effect (i.e., action versus no-action). Alc_NoAct = alcohol cues without drinking actions, Alc_Act = alcohol cues with drinking actions; Neu_NoAct = non-alcohol cues without drinking actions, Neu_Act = non-alcohol cues with drinking actions.

Next, the main effect of action versus no-action cues ((action alcohol + action non-alcohol) > (no-action alcohol + no-action non-alcohol)) was investigated. The results revealed two large significant clusters in the bilateral temporal lobe: the right inferior temporal gyrus, right middle temporal gyrus (x, y, z = 54, −57, −3; *t*(29) = 11.48, k = 994, FWE corrected *p* < 0.05), the left inferior temporal gyrus and left middle temporal gyrus (x, y, z = −54, −72, 0; *t*(1, 29) = 11.84, k = 1262, FWE corrected *p* < 0.05), and left parahippocampal gyrus and fusiform (x, y, z = −22 −46 −10; *t*(29) = −5.83, k = 74, FWE corrected *p* < 0.05) (Table 1, Figure 2).

#### 3.2.3. Interaction between Beverage Category and Cue Type

**Figure 3:**
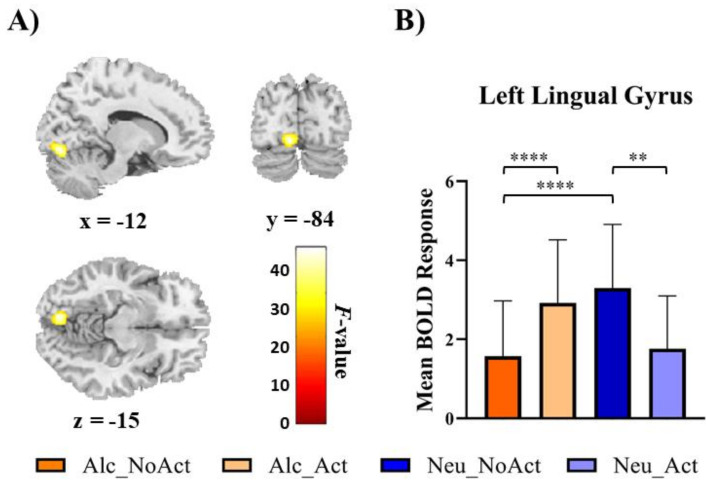
Neural interaction among cue category and cue type. (**A**) the group’s significant BOLD activity means the interaction between beverage type and action context; (**B**) mean BOLD response in the left lingual gyrus for each condition. Error bars show the associated beta values of the conditions in the significant cluster of the lingual gyrus. ** *p* < 0.01, **** *p* < 0.0001 after Bonferroni multiple-comparisons correction. Alc_NoAct = alcohol cues without drinking actions, Alc_Act = alcohol cues with drinking actions, Neu_NoAct = non-alcohol cues without drinking actions, Neu_Act = non-alcohol cues with drinking actions.

Finally, for the interactions between the factors of the beverage and the cue type, the repeated-measures ANOVA revealed a significant cluster in the left lingual gyrus region (x, y, z = −12, −84, −15; F(1, 29) = 52.94, k = 69, Figure 3A) that survived the FWE correction (*p* < 0.05). To explain the interaction effect in the left lingual gyrus, unplanned post hoc comparisons were conducted on the four possible experimental conditions (alcohol no-action, alcohol action, non-alcohol no-action, and non-alcohol action). It appeared that the interaction was mainly driven by a greater mean activation in alcohol no-action cues than alcohol action cues, *t*(29) = 7.28, *p* < 0.001, Bonferroni corrected (Figure 3B). In addition, no-action alcohol cues elicited less activation than their matched no-action non-alcohol cues, *t*(29) = 8.36, *p* < 0.001, Bonferroni corrected. Furthermore, for action cues, brain responses to alcohol cues were higher than those to matched non-alcohol cues, *t*(29) = 3.59, *p* < 0.007, Bonferroni corrected.

### 3.3. Relationship between Behavioral Data and fMRI Results

**Figure 4:**
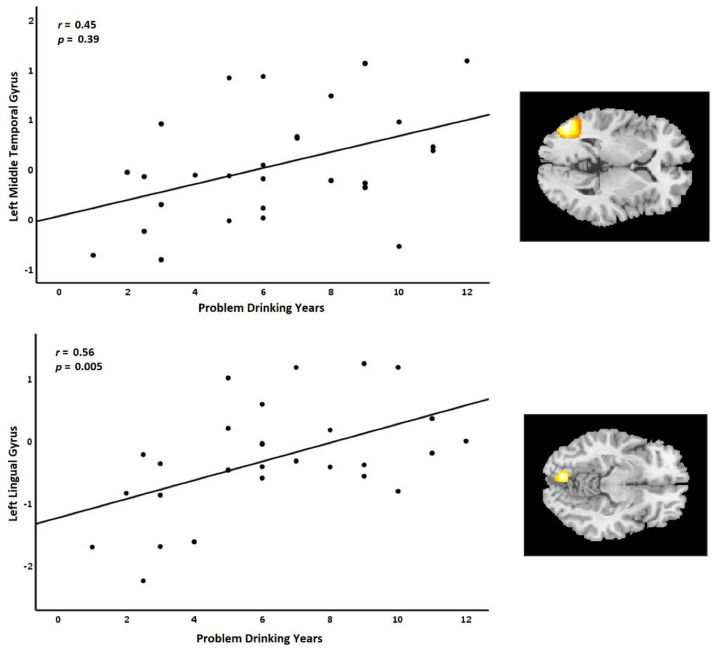
Positive correlations between the duration of problem drinking (years) and activation clusters were revealed by the whole-brain analysis in the left middle temporal gyrus and left lingual gyrus, elicited by the contrast of alcohol versus non-alcohol cues. Lines indicate linear associations; *r* = Pearson correlation; Alc = alcohol cue; Non-Alc = non-alcohol cue.

We analyzed the correlations between problem drinking years and the beta estimates of the action versus no-action and alcohol versus non-alcohol contrasts in brain activation revealed by the whole-brain analysis. Age was added as a control covariate. Problematic drinking years and mean BOLD response values (estimated from the contrast between alcoholic and non-alcoholic beverages) in the left middle temporal gyrus and left lingual gyrus were positively correlated and survived Bonferroni correction, *r*(29) = 0.45, *p* = 0.039, *r*(29) = 0.56, *p* = 0.005, respectively (Figure 4).

## 4. Discussion

The main aim of the current study was to compare brain activities to images of alcohol without any background (i.e., a beverage on a blank background) and those with associated human actions (i.e., a person interacting with the beverage). First, the whole-brain main effect of action versus no-action scenarios ((action alcohol + action non-alcohol) > (no-action alcohol + no-action non-alcohol)) revealed significant activations in the bilateral temporal lobe. Second, the whole-brain ANOVA results revealed a significant cluster in the left lingual gyrus region. Third, the correlation analysis between the contrast of alcohol and non-alcohol cues in the revealed clusters by the whole-brain analysis and the duration of problem drinking showed positive correlations in the left middle temporal gyrus and left lingual gyrus.

We expected that alcohol cues with action would activate sensorimotor regions in addition to the common cue-related regions. To see whether any brain regions match the hypothesis, repeated-measures ANOVA was performed. Indeed, we observed increased activations in the bilateral temporal lobe. Notably, temporal lobe activations were also reported in past studies that examined cue-reactivity effects in response to visual alcohol cues in people with AD (for review, see [35]). This finding partly verified our hypothesis. Similarly, other neuroimaging studies found a significant response to images of alcoholic drinks in the middle temporal lobe [36,37]. Areas associated with visual processing in the temporal and occipital lobes were also reported [38]. The temporal lobe activation has been associated with relational memory [39], which involves associative learning of multiple cues. Here, the activation might reflect a flexible use of learned information in response to alcohol cues [39]. As a result, our finding of the temporal lobe could represent a complex cue processing on multiple items rather than cue itself, which can indirectly support our notion that alcohol cues with actions can induce different cue-related brain regions. These explanations still require more studies to confirm.

Moreover, human drinking actions activate parts of the temporal cortex that are parts of the neural components of the default network. Activation in this area was suggested to participate in different component processes related to self-referential processing and memory-based scene construction, respectively [28]. This suggests that these regions involved in the utilization of self-referential processing and concurrent processes were linked with generating a mental scene based on memories, which were prominent in thinking about oneself in the future. Furthermore, our findings from human actions support, to a certain degree, a recent study investigating the induced activations of action representation using drug-use cues in heroin use subjects [13]. The study found that the bilateral middle temporal cortex showed significant activations toward the action cues.

Consequently, our results suggest that higher activations in response to cues with alcohol-related drinking actions might reflect complex cue processing or alcohol memory-related processing. In addition, the activations in the temporal regions may involve mixed tasks, such as response inhibition [40], which may contribute to relapse prevention treatment. However, the exact contribution requires further explorations in the future. Additionally, a possible explanation for the increased activation mainly in temporal areas and not in the frontal areas in the present study is that AD patients were more motivated to restrict their reactions to alcohol cues since they had just completed a phase of detoxification and strongly rejected alcohol-related signals. On the other hand, if AD patients were more motivated to resist alcohol signals, one may predict more activity in brain regions associated with cognitive control and not with impulsive control. This may also suggest that AD patients have a greater visual sensitivity to alcohol-related stimuli or have more difficulty discerning alcohol-related cues.

Furthermore, a substantial body of research exists explaining the involvement of dopamine (DA) in addiction, motivation, and reward-seeking behavior [41]. In addition to responses of dopamine to sensory stimuli, the rostromedial tegmental nucleus (RMTg) to dopamine neurons in the ventral tegmental area is involved in regulating cue-induced reward-seeking responses and punishment learning [42,43]. The principal role of the RMTg, a GABAergic midbrain region that records negative reward prediction mistakes and delivers dense inhibitory projections to midbrain dopamine neurons [43], is specifically to control reward-seeking in the presence of negative events. There is also a significant role of RMTg in punishment and avoidance learning, which shows that these neurons may store information about the stimuli encountered in these activities [44]. The learning patterns of these neurons are symmetrically opposed to those of dopamine firing. Inactivation of RMTg was found to increase alcohol seeking and consumption and improve cue-induced reinstatement of substance seeking [45]. Our results showed no activation in RMTg to alcohol cues. This may indicate that the recruited AD patients still have alcohol cravings, further supporting the relevance of reported activations in the bilateral temporal areas to the alcohol-related responses. Future neuroimaging studies in AD cue reactivity should study the role of RMTg in the responses to alcohol stimuli.

The interaction between beverage category and cue type analysis revealed that the lingual gyrus showed distinct activation. The literature on the neuroimaging of substance-related cue reactivity has been reported in the occipital cortex, which is still rarely treated as common regions of interest (ROIs) in addiction studies. However, previous meta-analyses studies have reported significant reactivity to substance-related cues in the occipital cortex [3,35,46]. Some human neuroimaging studies showed significant activations in the primary and secondary visual cortices when individuals with substance dependence were exposed to a drug cue (for a review, see [47]). Moreover, regions of the left occipital cortex, including the lingual gyrus, are associated with the recollection of memory [48] and craving level [49]. This suggests that activations of the occipital cortex may associate with memory-related activations. From the perspective of addiction, it is well established that people addicted to substances have a stronger attentional bias toward stimuli connected to their addiction [47]. The substance’s incentive salience transfers to the substance-related cue when the individual uses the substance regularly and finds it pleasurable, according to one theory of attentional bias in addiction. Occipital brain activity in response to substance cues may reflect an inherent sensitivity to substance signals rather than a developed attentional bias. Moreover, visual processing during observation of alcoholic stimuli is unquestionably more complex than processing simple stimuli and, hence, requires more resources in this area in general. This may indicate that certain regions are more active during brain response processes in response to alcohol-associated cues than they are in response to non-alcohol-associated cues. Therefore, the presence of a wide range of substance users emphasizes the consistency of visual cortex activation in the literature on substance addiction.

Furthermore, correlation analysis was performed between the duration of problem drinking as a measure of the severity of alcohol misuse and the contrasting activations elicited by alcohol and non-alcohol cues, and action and no-action cues. The analysis showed positive correlations between the duration of problem drinking and the contrasting activations elicited by alcohol and non-alcohol cues revealed by the whole-brain analysis in the left middle temporal gyrus and left lingual gyrus. The substance cue effect has been shown to associate with the duration of treatment [35]. This suggestion could be comparable to our results, as we also observed a correlation between dorsal striatum activation and the duration of problem drinking (Supplementary Materials; File S1). Finally, cue-reactivity effects may not always be consistent.

In addition, one of the main reasons behind the alcohol main effect results for the no-action condition is different from the existing literature results may be due to the type of recruited AD sample. This study may show a special situation of our sample, which is the patients with AD receiving treatment. Some of the patients had the motivation to suppress their cravings. Therefore, cues containing alcohol only with no human presence or drinking actions may not induce significant activations, compared with the cues containing drinking actions that may have increased incentive salience of alcohol cues that are more related to the drinking environment, which induced higher brain activity responses. Results of cue-reactivity-based fMRI studies are still inconclusive. The existing literature studies included original studies across different imaging modalities (fMRI, PET, and SPECT), approaches of fMRI data preprocessing (whole brain and ROI methods), samples (current outpatient treatment, recent inpatient detoxification, treatment and non-treatment seeking, and placebo treated), cue type (visual, taste related, and olfactory), and visual alcohol and control cues (alcohol with/without human presence, shapes, food, disgust-related objects, and household objects). Therefore, the reasons above may also explain the difficulty of replicating existing responses to no-action alcohol cues. Additionally, future research with larger sample size is needed to investigate if brain BOLD activity can distinguish between BOLD patterns triggered by the presence of a human with beverage interactions toward alcoholic and non-alcoholic drinks in AD patients.

The present study has some limitations. First, our sample size was small but still reasonable; future studies should include more participants and a control group for more robust results and validation. Second, very few women in China suffer from substance use disorders [50]. Therefore, only male patients were included. Although previous studies found no gender effect on the brain responses to alcohol cues [51,52], future studies should include female ADs. Third, our sample was AD inpatients who had different abstinence days at treatment initiation. This may affect the relationship between this duration and prefrontal–striatal functional pathology [53]. Forth, AUDIT was used as an extra measurement for assessing the AD severity, which may not be the ideal instrument for assessing AD severity. However, the AUDIT assesses the history of alcohol addiction that reflects the history of patients’ alcohol problem consumption, drinking behaviors, and alcohol-related problems, which we believe can indicate alcohol use severity. In addition, the AUDIT was used following previous studies (e.g., [33,51,54]). Finally, because we only employed no-action cues with no human presence in the background, it is possible that we may not rule out the effect of the human presence-specific cue from the action-specific cue. The statistical power for evaluating no-action alcohol brain activity might be restricted due to the small sample size of subjects, which is still reasonable (Supplementary Materials; File S1).

## 5. Conclusions

The present findings support previous substance cue-reactivity studies involving the effects of action cues in drug-induced reactivity [13,21,55]. The current study shows that the action-related cues to alcohol use activate the corresponding sensorimotor system and self-referential processing regions, such as temporal cortex regions and lingual gyrus. The observed results can help understand the habitual behavior of alcohol use and the role of action-related cues for alcohol use in predicting the alcohol cue-reactivity effects. We recommend that studies focusing on alcohol cue reactivity carefully consider cues depicting human drinking actions, as our results indicate that some brain regions other than reward-related regions are also involved. The present study’s findings point to the possibility of using the sensorimotor system cortical representation impact in cue exposure treatment for alcohol addiction.

## Figures and Tables

**Table 1:** Significant clusters of cue type main effect by contrasting action versus no-action cues.

Anatomical Location	Hemisphere	Peak	Cluster Significance *p*-Value	Cluster Size k	*x*	*y*	*z*
Contrast: Action > No-Action							
ITG, MTG, and MOG	R	11.48	0.000	994	54	−57	−3
ITG, MTG, and MOG	L	11.84	0.000	1262	−54	−72	0
PG, FF	L	−5.83	0.000	74	−22	−46	−10

**All coordinates are provided in MNI Space.** ITG, inferior temporal gyrus; MTG, middle temporal gyrus; MOG, middle occipital gyrus; PG, parahippocampal Gyrus; FF, fusiform; R, right; L, left.

## Data Availability

Data are available upon reasonable request to the corresponding author.

## Supplementary Materials

The following are available online at https://www.mdpi.com/article/10.3390/brainsci12030307/s1, Table S1: The demography data of the participants, Table S2: A brief list of inclusion and exclusion criteria., Table S3: Examples of alcoholic and non-alcoholic beverages are presented in scenarios, Table S4: Comparison of craving scores after the presentation of action and no-action cues., Table S5: Whole-brain activations wherein alcohol-related pictures elicited higher activations than matched non-alcohol-related picture, Figure S1: Power analysis of cue set of a single condition consists of 20 cues, Figure S2: This plot shows the average subjective craving scores compared with the type of drink (i.e., alcohol and non-alcohol), Figure S3: Average subjective craving scores, Figure S4: Whole-brain activations wherein alcohol-related pictures elicited higher activations than matched non-alcohol-related pictures, Figure S5: Increased (red) BOLD signal of the contrast of alcohol versus non-alcohol, Figure S6: One-sample *t*-test for action and no-action conditions with problematic drinking years as covariate of no interest. Figure S7. Positive correlations between the duration of problem drinking (years) and activation of the dorsal striatum, Table S1: The demography data of the participants Table S2: A brief list of inclusion and exclusion criteria, Table S3: Examples of alcoholic and non-alcoholic beverages are presented in scenarios., Table S4: Comparison of craving scores after the presentation of action and no-action cues, Table S5: Whole-brain activations wherein alcohol-related pictures elicited higher activations than matched non-alcohol-related pictures.
