# Supplementary material for: Depicting People in Visual Cues Affects Alcohol Cue Reactivity in Male Alcohol-Dependent Patients
Source: Brain Sci. 2022 Feb 24;12(3):307. doi: 10.3390/brainsci12030307 (PMC8946691; doi:10.3390/brainsci12030307)
Supplement: Supplementary file 1 [file brainsci-12-00307-s001.zip › brainsci-1495457-supplementary.pdf]

Supplementary Materials

1. Materials and Methods

1.1 Subject Details

All of the participants were enrolled from Anhui Mental Health Center, a leading provider of alcohol rehab programs in Anhui Province in China. An inpatient 42-day rehab program for AD was provided. It contained numerous pharmacological treatments and psychosocial treatments. During the first week after admission, diazepam was initially given at a dose of 20 mg intravenously twice a day, for 3-4 days, to smooth the course of withdrawal in case of severe alcohol withdrawal syndrome (SAWS) such as delirium tremens and/or seizures. After that, the diazepam dose was decreased gradually until discontinued within another 3-4 days. Moreover, hepatic function damage was provided for the necessary medical management of physical diseases, such as electrolyte imbalance. Starting from the second week after admission, a small dose of quetiapine was given at a maximum dose of 200 mg, to reduce the craving for alcohol and prevent long-term relapse.

Meanwhile, various types of behavioral therapies were used, such as motivational interviewing, brief intervention, and cognitive behavioral therapy. The whole program lasted for 42 days, and all participants in the sample were exposed to the same treatments. Before the experiment, patients were abstinent from alcohol for 2–3 weeks (mean, 25 days; range, 15–44 days) and were not allowed to consume alcohol in the center. In addition, necessary rehabilitation treatment was taken for alleviating withdrawal symptoms. The treatment included psychological support, psychoeducation, and medical management of physical diseases, such as electrolyte imbalance, hepatic function damage. Moreover, all patients received a small dose of quetiapine treatment. Patients with any neurological or mental disorders or dependent on substances other than alcohol or nicotine were not recruited. Tables S1 and S2 show more demographic data and the inclusion/exclusion criteria details.

The duration of problem drinking was assessed in the present study. However, there are not many details reported about this item's validity or reliability. Generally, problem drinking is associated with losing control over one's alcohol intake and/or displaying signs that alcohol consumption is interfering with the AD patient's life activities. In terms of the DSM-5 factors discussed, the person would begin to show more symptoms in such a case. At this stage, a person is physically dependent on alcohol. The duration of problem drinking has generally been considered the crucial variable in alcoholics' impaired neuropsychological performance. It is generally known that those addicted to substances have a higher attentional bias to substance-related stimuli. At the stage of severe substance dependence, apart from external cues, behavior in this advanced stage of dependence is governed by internal (withdrawal) cues (Vollstädt-Klein et al. 2011) or habit (Everitt and Robbins 2005), which support studying the correlation between problematic drinking duration and the response to alcohol cues. Therefore, the duration of problem drinking was used to assess participants' potentially problematic drinking behavior duration. The participants were aware of the meaning of the problematic drinking duration, which means the duration of irregular drinking patterns (i.e., not on specific daytime) with different amounts of alcohol and reflects harmful drinking patterns. To confirm the reliability of the scores, one or more family members of each participant were asked the same question at the admission time of the treatment program. Selecting this item was influenced by a study led by Joos et al. (2012) that showed that clinical characteristics and neuropsychological variables contributed independently to the age of onset of problematic alcohol use. In addition, their results indicated that especially an impulsive reflection style, in addition to higher trait impulsivity, may be the core feature of early onset alcohol dependence.

Table S1. The demography data of the participants (n = 30).

Items	*M*	*SD*
**Demographics**		
Age	39.63	8.15
Right handed, N = 30	-	-
Years of education	5.07	1.08
**Clinical characteristics**		
Depression severity (BDI)	8.92	6.43
Anxiety severity (BAI)	25.00	3.94
AUDIT	26.20	6.70
Years of problematic drinking	12.43	3.13
Years of drinking	19.23	7.51
Duration of AD (years)	15.07	11.56
Consumption (grams/day) (Mdn = 1000 (150-6000))	1084.58	1004.18
Abstinence (days)	25.12	7.73
Treatment duration (days)	47.12	31.40
Smokers, N = 30	-	-
FTND	5.85	1.88
PANAS (positive emotion score)	24.20	7.28
PANAS (negative emotion score)	15.92	4.33
**Behavioral characteristics**		
PACS (Craving before fMRI task)	6.20	6.97

SD = standard deviation; Mdn = median; FTND = Fagerstrom Test for Nicotine Dependence; PANAS = positive and negative affect schedule; PACS = Pennsylvania Alcohol Craving Scale.

Table S2. A brief list of inclusion and exclusion criteria.

No.	Inclusion	Exclusion
**1.**	18-55 years old, Han nationality, right-handed	Meet the DSM-IV diagnostic criteria for other mental disorders (except for nicotine dependence)
**2.**	Meet the DSM-IV diagnostic criteria for alcohol dependence, without acute alcohol withdrawal syndrome (stop drinking for 14-30 days), and have a CIWA-Ar score < 7	Complicated with insufficiency or failure of important organs such as heart, liver, lung, kidney, and brain
**3.**	Normal vision or corrected vision	Taking drugs with significant anticholinergic effects, such as clozapine,

		olanzapine, chlorpromazine, perphenazine, etc.
**4.**	Blood test: negative for ethanol and benzodiazepines	Taking drugs that significantly affect alcohol cravings or impulsivity for a long time 1 month before enrolment or during follow-up
**5.**	Be able to write and read Chinese, understand informed consent, and have no cognitive impairment or mental development problems	Subjects with a history of brain injury
**6.**	Have no implanted medical devices or other clinical contraindications to scanning, such as fear of closed places.	Subjects with poor compliance and fail to complete the experiment
**7.**		Subjects who quit or with adverse events

DSM = Diagnostic and Statistical Manual of Mental Disorders; CIWA-Ar = Clinical Institute Withdrawal Assessment Alcohol Scale-Revised.

1.2. Cue Design

1.2.1. Overview

This supplementary report provides additional information about the cue design of our cue set and more details about the matching issue of alcohol and non-alcohol with action/no-action.

1.2.2. Subjects

In total, 22 participants with AD (mean age, 40.2 years with an age range of 25 to 56 years) were recruited from Anhui Mental Health Centre at Hefei Fourth People's Hospital. All participants were males due to the very low prevalence (only 0.1%) of female ADs in China (Cheng et al. 2015). At the time of the study, all patients were under an inpatient treatment protocol. All patients agreed to participate in the experiment and met the criteria for alcohol dependence syndrome as their primary diagnosis, according to the International Classification of Disease, 10th edition (ICD-10: F102).

1.2.3. Cues Production

Both alcohol and non-alcohol cue pictures were first selected based on famous brands and beverages in China. These pictures were obtained from open websites and shot by the investigators. In general, alcohol-related pictures are alcoholic beverages in glasses or bottles of wine/beer in different alcohol-related scenarios. Given that alcohol and non-alcohol stimuli differ in many aspects, such as overall attractiveness, matched non-alcoholic beverages pictures were used as a control condition, thereby decreasing confounding factors in the contrast of alcohol versus control incentives.

This study mainly focused on studying the brain responses to the human presence with drinking actions in alcohol cues, which requires action cues to view a human interacting with the drink. Studying combined human presence with the interaction with the beverage container would need to contrast action cues with human presence and no-action cues with no human presence. Additionally, the used cues in our study did not include no-action cues with human presence due to some concerns. The patients' sample was undergoing AD treatment and were trained to suppress their cravings. In addition, we believe seeing the no-action cues with human presence but not drinking it or interacting with it may imply to the patient inhibit your (his) craving to these cues. Therefore, due

to the explained reasons, these cues were not included in this study. We recommend that future studies should include such cues.

1.2.3.1. Categories and Contexts of Pictures

1.2.3.1.1. Beverage Types and Subtypes

The first picture selection process ended with around 300 alcohol pictures, and all went through the second step of the rating process. A total of 40 alcohol and 40 matched non-alcohol images with/without human presence were selected as a cue set for the cue-reactivity experiment. The following types and subtypes of pictures were included:

- Alcoholic beverages were beer, white wine, red wine, and yellow wine;
- Non-alcoholic beverages were water, soft drinks, and miscellaneous.

In addition, a power analysis was performed using the G*Power software to check if the sample size of the cues set selected for cue reactivity was sufficient. Figure S1 shows that the power of the cues' sample size was still reasonable.

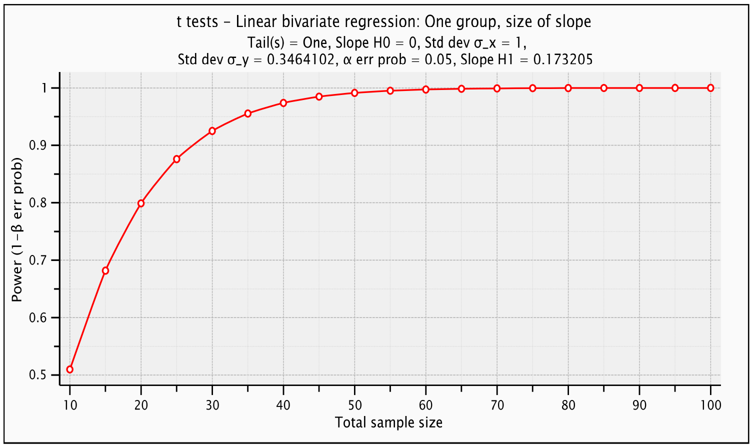

Figure S1. Power analysis of cue set of a single condition consists of 20 cues.

1.2.3.1.2. Contexts

Each category was collected and photographed in two no-action scenarios (i.e., unopened bottles and full glasses with blank background), in which a beverage was presented without any human body parts, and three action scenarios (i.e., opening bottle, pouring a drink, and drinking), in which an actor operated on a beverage.

1.2.4. Controlled Variables

The pictorial stimuli were matched concerning shooting scenarios (i.e., bottle with bland background, opening a bottle, pouring a drink, and drinking) and objective measures of brightness level. These aims were accomplished by (1) pairing each alcohol picture with a non-alcohol picture of another scene as closely as possible for content; (2) matching approximately each alcohol picture to a neutral picture for color, form, angle of action; (3) obtaining the objective measurement of pictures' brightness and complexity. For each image, we computed relevant image properties that characterize the images' physical appearance. The following image properties were analyzed:

- Brightness: We made brightness adjustments when stimuli required it;

- Color: quantified as the proportional contribution of the red, green, and blue channel, averaged across all non-white pixels;
- Size of pictures: The pictures in the stimulus set were 700 by 450 pixels;
- Framing of action pictures: In order to avoid probable confounding effects in pictures, we only allowed the necessary parts of actors' bodies to enter the frame. Hence, human faces were removed to control other potential confounding effects. However, due to some pictures involving the factor of actions, faces were preserved to avoid looking unnatural (i.e., hands holding drinks or raising them to the mouth with no full picture of a face);
- Matching types of beverages: The alcohol and non-alcohol with action/no-action were paired and matched approximately each alcohol picture to a neutral picture for color, form, angle of the action.

1.2.4.1. Backgrounds

Backgrounds were set up to be as bland as possible (i.e., having a minimum of features).

Table S3. Examples of alcoholic and non-alcoholic beverages are presented in scenarios.

Context	Sub-context	Alcohol	Non-Alcohol
No-Action Scenarios	Beverage in bottle	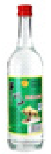	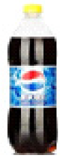
Action Scenarios	Opening a bottle	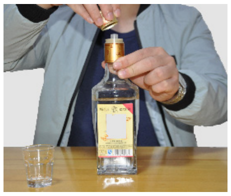	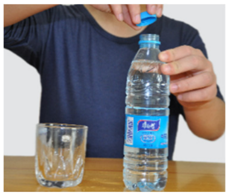
	Pouring a beverage	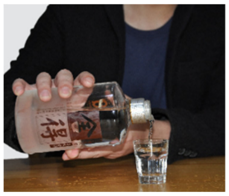	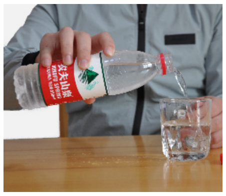
	Consuming a beverage	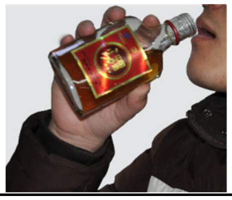	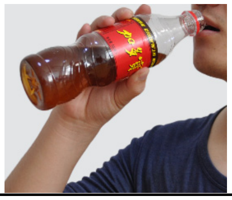

1.2.5. Cue Set Scores

No-action alcohol cues each depicted a simple image of an alcoholic beverage. No-action non-alcohol cues contained a matched non-alcoholic beverage (e.g., a bottle of wine in a no-action alcohol cue was matched with a bottle of soft drink in a no-action non-alcohol cue). Action cues (alcohol and non-alcohol) were similarly matched. Still, they

showed each beverage in the context of a person interacting with it, e.g., opening a bottle, pouring a drink, or drinking (Figure S2). The craving scored for the alcohol and non-alcohol cues during the rating procedure, with 5 as the maximum score. To compare craving scores after the presentation of action and no-action cues, paired-sample *t*-tests were conducted. No differences were observed between craving scores for alcohol action cues (M = 3.40, SD = .42) and alcohol no-action cues (M = 3.01, SD = 1.09), $t(19) = 1.52$, $p = .071$, or between craving scores for non-alcohol action cues (M = .82, SD = .31) and non-alcohol no-action cues (M = .66, SD = .56), $t(21) = 1.26$, $p = .110$. In addition, the results of the rating procedure showed a significant difference of craving scores between alcohol (M = 3.36, SD = .47) and non-alcohol pictures (M = .86, SD = .39), $t(78) = 24.24$, $p < .001$. The supplementary figures show the subjective craving scores differences of the types of pictures (Table S4, Figures S2 and S3).

Table S4. Comparison of craving scores after the presentation of action and no-action cues.

Region	Action		No-action		*t*	*p*
	M	*SD*	*M*	*SD*		
Alcohol	3.40	.42	3.01	1.09	1.52	.071
Non-alcohol	.82	.31	.66	.56	1.26	.110

M = mean. SD = standard deviation.

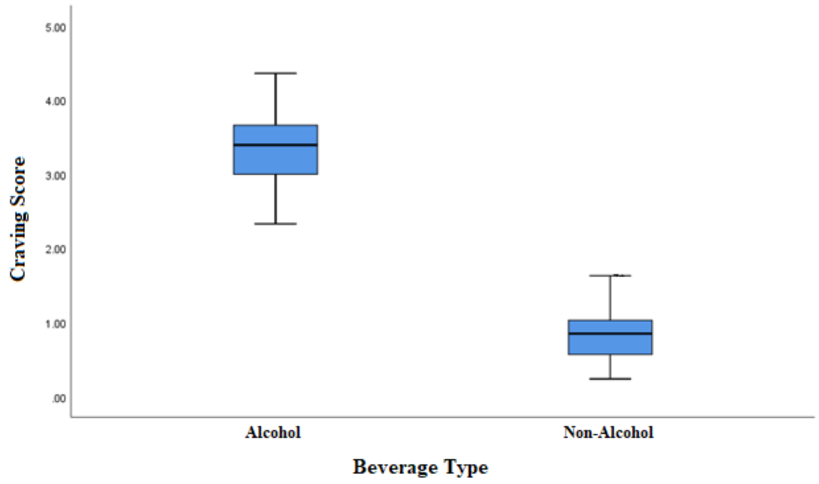

Figure S2. This plot shows the average subjective craving scores compared with the type of drink (i.e., alcohol and non-alcohol).

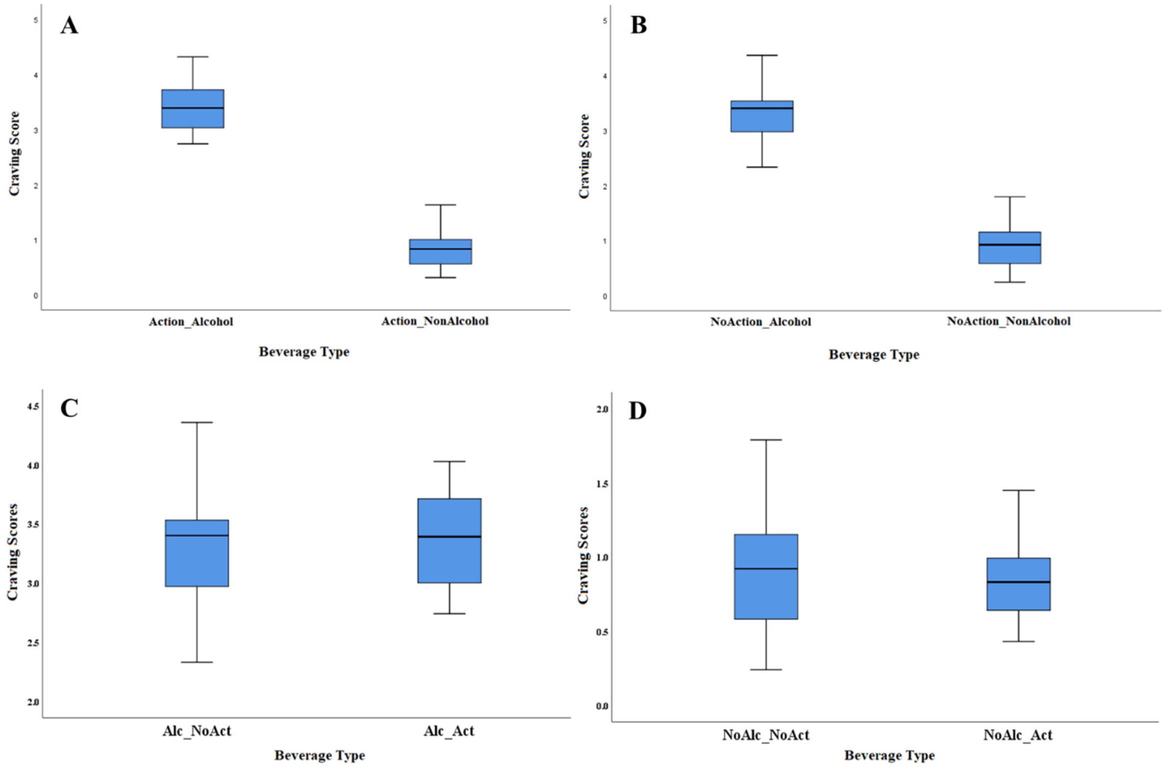

Figure S3. (**A**) This plot shows the average subjective craving scores, compared with action context for two types of beverages (i.e., alcohol and non-alcohol); B) this plot shows the average subjective craving scores, compared with no-action context for two types of beverages (i.e., alcohol and non-alcohol); (**C**) this plot shows the average subjective craving scores compared with alcohol cues for two types of actions context (i.e., action and no-action). Alc = alcohol; Non-Alc = non-alcohol. Act= action; NoAct= no-action; (D) this plot shows the average subjective craving scores, compared with non-alcohol cues for two types of actions context (i.e., action and no-action). Alc = alcohol; Non-Alc = non-alcohol. Act= action; NoAct= no-action.

1.3 A Priori ROIs for Correlation Analysis

Pearson correlations were also performed between the problem drinking years and the mean response in specific brain regions, including ventral tegmental area, ventral striatum, dorsal striatum, orbitofrontal cortex, anterior cingulate cortex, and dorsolateral prefrontal cortex. Since we did not find strong activation in reward-related regions, the above regions were selected based on the alcohol literature [1]. These brain regions were previously reported to show positive correlations with alcohol dependence and severity [2, 3]. The anatomical masks of the prior regions of interest (ROIs) were created using the WFU_PickAtlas toolbox (https://www.nitrc.org/projects/wfu_pickatlas) for the bilateral ROIs, in which six masks in total were created. Then, the averaged BOLD signal (beta values) from the alcohol versus matched non-alcohol and action versus no-action contrasts were extracted per individual using MarsBaR (http://marsbar.sourceforge.net/). Bonferroni correction was applied for multiple comparisons.

2. Results

2.1. fMRI Results

2.1.1. Main Effect of Category Types

Parameter estimates under the four experimental conditions were extracted from the activated clusters and submitted to a two (cue category) by two (cue type) repeated-measures ANOVA. We identified the brain regions associated with the main effect of cue category (i.e., type of beverage). We only found decreased activations in a cluster lying in the right lingual gyrus that survived the FWE correction (x, y, z = 13, -77, -7; $t(29) = -5.93$, k = 66, FWE correction $p < .05$) (Figure S4). When applying significance at $p < .001$ (uncorrected), we found only deactivations in two clusters including the right lingual gyrus (x, y, z = 12, -78, -6; $t(29) = -5.93$, k = 3642, $p < .001$ (uncorrected)) and the left fusiform (x, y, z = -28, -56, -12; $t(29) = -3.91$, k = 103, $p < .001$ (uncorrected)). Moreover, we investigated another level of contrast, which is the alcohol cue reactivity effect by extracting two experimental conditions (i.e., the contrast of alcohol > matched non-alcohol; Figure S5 and Table S5).

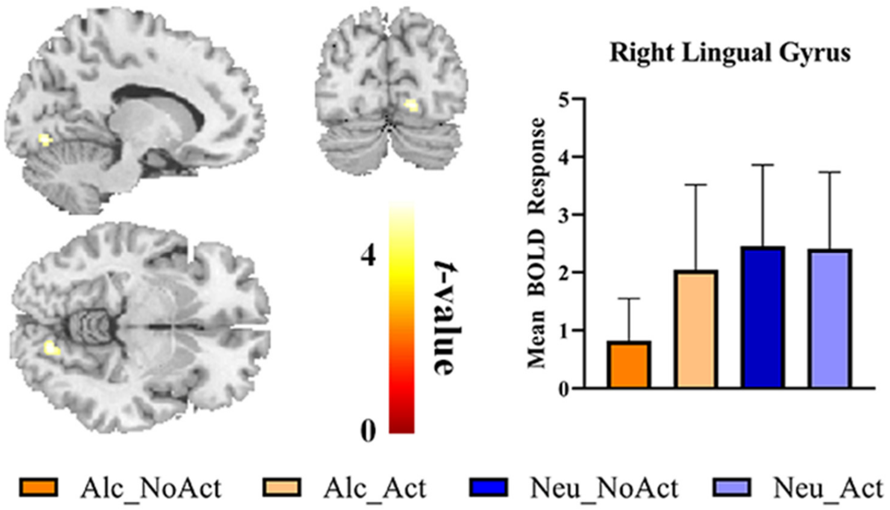

Figure S4. Whole-brain activations wherein alcohol-related pictures elicited higher activations than matched non-alcohol-related pictures **4.** Brain activations were elicited by visual stimuli that indicate alcohol's main effect. Alc_NoAct = alcohol cues without drinking actions, Alc_Act = alcohol cues with drinking actions, Neu_NoAct = non-alcohol cues without drinking actions, Neu_Act = non-alcohol cues with drinking actions.

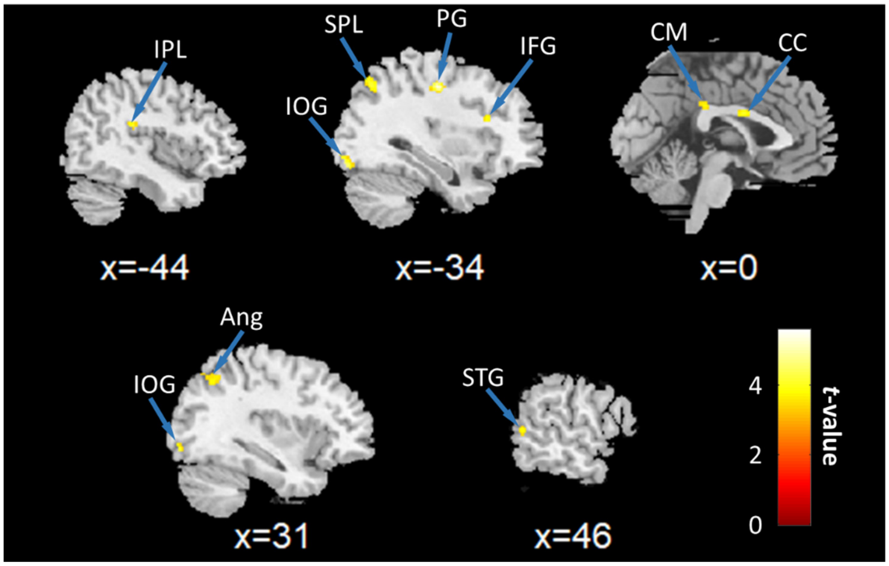

Figure S5. Increased (red) BOLD signal of the contrast of alcohol versus non-alcohol ($p < .001$ uncorrected). IOG, inferior occipital gyrus; MTG, middle temporal gyrus; IPL, inferior parietal lobule; IFG, inferior frontal gyrus; CC, corpus callosum; CM, cingulum middle; SPL, superior parietal lobule; Ang, angular; PG, precentral gyrus.

Table S5. Whole-brain activations wherein alcohol-related pictures elicited higher activations than matched non-alcohol-related pictures (p <.001, uncorrected).

Region	sphere	*t*-value	Cluster significance *p-value*	Cluster size k	*x*	*y*	*z*
Contrast: *Alcohol* > *Non-alcohol*							
MTG	R	5.23	.000	45	28	-90	-8
IPL	R	3.77	.000	19	60	-58	8
IFG	L	3.85	.000	15	-34	20	22
CC	L	4.19	.000	37	0	0	26
PCC		4.00	.000	17	0	-30	32
SPL	L	3.84	.000	21	-32	-70	50
Ang	L	4.17	.000	91	36	-62	44
PG	R	3.78	.000	32	-34	-18	46
MFG	R	3.87	.000	39	46	28	25
IOG	R	5.57	.000	44	83	-90	-8
IOG	L	5.51	.000	100	-28	-92	-10

IOG, inferior occipital gyrus; MTG, middle temporal gyrus; IPL, inferior parietal lobule; IFG, inferior frontal gyrus; CC, corpus callosum; PCC, posterior cingulum cortex; SPL, superior parietal lobule; Ang,

angular; PG, precentral gyrus; MFG, middle frontal gyrus. Coordinates of brain regions are reported in MNI coordinates. L, left; R, right; uncorr, uncorrected; MNI, Montreal Neurological Institute.

2.2 Regression with Problematic Drinking Years

To calculate a regression with problematic drinking years as a covariate of no interests in order to evaluate if the conditions are the same, a group statistic (one-sample *t*-test) for action and no-action was performed (Figure S6). The contrast alcohol action > no alcohol action of group level (one-sample *t*-test) with problematic drinking years as a covariate of no interest showed increases in multiple brain regions at $p < .001$ (uncorrected). The contrast alcohol no action > no alcohol no action of group level (one-sample *t*-test) with problematic drinking years as a covariate of no interest showed decreases mainly in the occipital lobe at $p < .001$ (uncorrected).

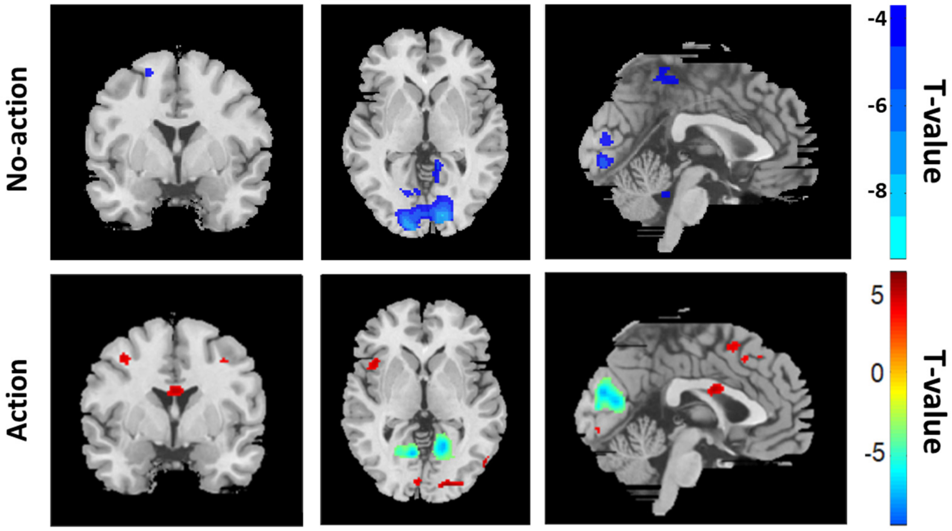

Figure S6. One-sample *t*-test for action and no-action conditions with problematic drinking years as covariate of no interest. No-action = no-action cues (alcohol > non-alcohol), Action = action cues (alcohol > non-alcohol).

2.3. Relationship between Behavioral Data and fMRI Results

We also analyzed the correlations between problem drinking years and the beta estimates of the action vs. no-action and alcohol vs. non-alcohol contrasts in a priori ROIs, including anterior cingulate cortex, dorsolateral prefrontal cortex, dorsal striatum, orbitofrontal cortex, ventral tegmental area, and ventral striatum. Problem drinking years and mean BOLD response values (estimated from the alcohol vs. non-alcohol beverages contrast) only in the dorsal striatum, was positively correlated and survived Bonferroni correction, r(29) = .51, p = .018 (Figure S5). Age was added as a control covariate.

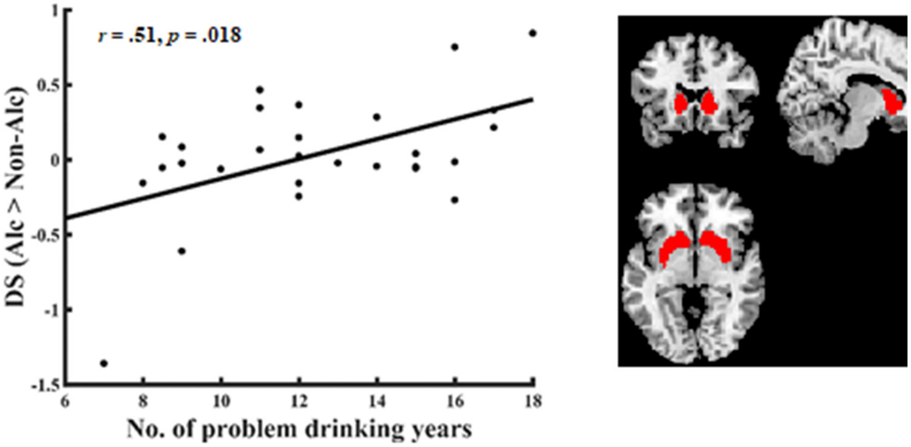

Figure S7. Positive correlations between the duration of problem drinking (years) and activation of the dorsal striatum (DS), elicited by the contrast of alcohol versus non-alcohol cues. Lines indicate linear associations; r = Pearson correlation; Alc = alcohol cue; Non-Alc = non-alcohol cue.
